# Problems and challenges encountered by Chinese medical institutions in implementing the national centralized drug procurement

**DOI:** 10.3389/fphar.2023.1233491

**Published:** 2023-08-30

**Authors:** Wen Zhang, Qingwen Xu, Jing Peng, Xiaotong Zhang, Lu Chen, Yilai Wu, Kui Yang, Jiajie Luan, Xiaoyun Liu

**Affiliations:** The First Affiliated Hospital of Wannan Medical College (Yijishan Hospital of Wannan Medical College), Wuhu, Anhui, China

**Keywords:** national centralized drug procurement, medical institutions, problems, challenge, China

## Abstract

**Objective:** The problems and challenges encountered by Chinese medical institutions in implementing the national centralized drug procurement was investigated and analyzed in order to provide reference for the regulatory agencies to formulate policies.

**Methods:** A questionnaire survey was conducted to collect the problems encountered by 329 Chinese medical institutions in implementing the national centralized drug procurement and the corresponding suggestions provided by relevant experts. Statistical analysis was performed to identify differences in the themes and the number of collected problems, further revealing the relevance to the region in which the medical institutions is located.

**Result:** 1360 problems and suggestions were collected from 329 Chinese medical institutions that located in North (19.15%), Northeast (5.78%), East (33.43%), Central (10.03%), South (9.73%), Southwest (14.89%), and Northwest China (6.99%). There was statistically significant difference in the number of collected problems and suggestions between regions (*p* < 0.001). Furthermore, the content of gathered problems and suggestions involves in 15 themes including system construction, organizational system and work responsibilities, reasonable measurement and reporting of procurement volume et al. These themes that these medical institutions are focusing on are mainly centered on the supply guarantee (15%), reasonable measurement and reporting of procurement volume (11.40%) and guarantee measures for clinical priority use (9.48%) of drugs with national centralized procurement. Meanwhile, we found that problems regarding the supply guarantee of drugs with national centralized procurement displayed significant difference between regions (*p* = 0.0096).

**Conclusion:** Chinese medical institutions are facing great challenges in implementing the national centralized drug procurement. The scientific study and judgment of the current situation and the construction of corresponding solution require a precise classification of the problems encountered by medical institutions in the process of implementing the national centralized drug procurement policy, which is of great practical significance for deepening the reform of the medical and health system.

## Introduction

Increasing pharmaceutical expenditures is plaguing many countries worldwide (Lopez Bastida et al., 2000; Smith.2004). Global medicine spending is projected to increase at 2–5% annually and exceed $1.1 trillion in 2024 (Institute.2020). In China, healthcare expenditures increased rapidly at a nearly 20% annual growth rate (Yuan et al., 2021). In order to reduce the pharmaceutical expenditures and standardize drug circulation, the Chinese government has implemented national centralized drug procurement (NCDP) since 2018. As of February 2022, prices of 234 medicines have been lowered through national centralized procurement, with the average rate of reduction exceeding 53%, and the cumulative savings in drug costs have exceeded 260 billion yuan (Zou et al., 2023). Moreover, national centralized drug procurement is being optimized and improved, gradually including the basic medical insurance drugs with bulk usage and procurement-volume and all types of clinical essential medicines with reliable quality (Chinese Government.2019; 2021).

Public medical institutions are required to participate in the national centralized drug procurement and prioritize the use of drugs with national centralized procurement (DNCP) in accordance with clinical demands (Gong et al., 2021). The application of NCDP has brought significant economic and social benefits and enhanced the rational use of clinical drugs, playing a positive effect on healthcare reform in China (Chen et al., 2020; Yuan et al., 2021). For instance, pharmaceutical expenditures and irrational clinical use rate were reduced comparison before and after NCDP policy (Hu et al., 2022; Wan et al., 2022; Wang et al., 2021). Meanwhile, drug utilization and substitution rate of generic drugs significantly increased after policy intervention (Xie et al., 2021; Zhao et al., 2022). However, some problems have been disclosed in practicing NCDP by medical institutions, which significantly affected the effects of NCDP implementation. For example, lacking scientific estimation system resulted in large discrepancies between procurement volume and usage volume by medical institutions (Xu.2022). Due to the difficulty of securing drug supply in primary medical institutions, some drugs with national centralized procurement are in shortage or even out of supply, thus affecting clinical use of drugs (Guo et al., 2015). In some medical institutions, non-winning drugs are completely discontinued in order to complete the contract dosage, so that the patient’s drug needs are not met, thus aggravating the doctor-patient disputes (Li et al., 2022). Therefore, it is necessary and urgent to comprehensively understand the difficulties encountered by medical institutions in implementing the NCDP and provide corresponding solutions to promote the in-depth implementation of the national centralized drug procurement policy.

To further improve the standardization of NCDP policy at medical institution level, several provinces and autonomous regions have issued expert consensus or recommendations on the implementation of NCDP in Chinese medical institutions (Chen et al., 2022; Ye et al., 2021). Our group as co-leader gathered up 302 multidisciplinary experts from 128 Chinese medical institutions to write the “ Consensus of Chinese expert on the precise management of national centralized drug volume-based procurement in medical institutions” in 2022 (Chinese Pharmacists Association.2023). This study aims to comprehensively reveal the problems encountered by Chinese medical institutions during implementing of NCDP policy and provide relevant recommendations given by experts, which is an important reference for the development of relevant policies.

## Methods

### Research subjects

This study conducted a targeted survey of 329 Chinese public medical institutions from 22 provinces, 5 autonomous regions and 4 municipalities directly under the Central Government (excluding Hong Kong, Macao and Taiwan). These medical institutions include provincial or university-affiliated hospitals, municipal, county and community health service centers. Meanwhile, 578 participants, which belong to multiple disciplines or departments including pharmacy department, medical department, clinical department, medical insurance department, and medical quality control department, involved in this survey.

### Questionnaire design and data collection

After considerable discussion, the outline of the questionnaire was determined by the research committee. As is shown in Supplementary Table S1, the questionnaire contains 6 primary catalogs which further subdivided into 15 secondary catalogs, involved the whole process of implementation of NCDP in medical institutions. Besides, open-ended questions included in each catalog intentionally, mainly for collection of the problems in the implementation of NCDP and corresponding suggestions. Subsequently, the questionnaires were directly conducted to 329 medical institutions. These collected questions are categorized into 15 secondary categories. Regarding the handling of open-ended questions, they will be grouped under the 15 entries mentioned above. However, in case of disputes they will be further discussed by the research committee. Ultimately, these questionnaire results were further analyzed, summarized, and organized using statistical methods.

### Statistical methods

IBM 24.0 SPSS statistical software (IBM Corp., Armonk, NY, United States) was used for analysis. Count data were expressed as cases or percentages, and the χ^2^ test and one-way ANOVA analysis were used for comparison between groups. *p* < 0.05 was considered a statistically significant difference.

## Results

### Characteristics of the surveyed medical institutions and respondents

To examine the status of implementing of national centralized drugs procurement in Chinese medical institutions, this study conducted targeted research on 329 Chinese medical institutions. The recall rate and efficiency rate of the questionnaires were 100% and 100%, respectively, attributing to the fact that the research was conducted in a targeted manner. The regional and hierarchical distribution of medical institutions were taken into full consideration. As shown in Table 1, the regions of 329 surveyed medical institutions were located North (19.15%), Northeast (5.78%), East (33.43%), Central (10.03%), South (9.73%), Southwest (14.89%), and Northwest China (6.99%). These investigated institutions contain the provincial or university-affiliated medical institutions (52.28%), municipal medical institutions (31.31%), community health service centers (13.98%), and county-level medical institutions (2.43%). Of note, a total of 578 participants mainly distributed in department of pharmacy, medical, clinical, medical insurance, and medical treatment control, basically covering the essential departments for implementing national centralized drugs procurement. Among of these participants, pharmacy department presented largest number of personnel (89.45%) (Table 2), because they have taken on the primary task of implementing NCDP in medical institutions. Taken together, this research not only reflects the actual situation of implementing of NCDP in different regions and levels of Chinese medical institutions, but also visualizes the problems encountered by the main enforcement departments of NCDP in medical institutions.

**TABLE 1:** Characteristics of the medical institutions.

Characteristic	Number (%) *n* = 329
**Regions**	
East China	110 (33.43)
North China	63 (19.15)
Southwest China	49 (14.89)
Central China	33 (10.03)
South China	32 (9.73)
Northwest China	23 (6.99)
Northeast China	19 (5.78)
**Types**	
Provincial or university -affiliated medical institutions	172 (52.28)
Municipal medical institutions	103 (31.31)
Community health service center	46 (13.98)
County-level medical institutions	8 (2.43)
**Levels**	
Comprehensive medical institutions	311 (94.53)
Specialized medical institutions	18 (5.47)

**TABLE 2:** Distribution of the departments of the participants.

Discipline or department	Number of staff	Percentage (%)
Pharmacy Department	517	89.45
Medical Departments	36	6.23
Clinical Departments	20	3.46
Medical Insurance Department	3	0.52
Medical Quality Control Department	2	0.35

### Characteristics of problems encountered by Chinese medical institutions in implementing of NCDP

In order to gain an in-depth understanding of the difficulties faced by Chinese medical institutions in implementation of national centralized drug procurement, the 1360 gathered problems and suggestions were further analyzed. As is shown in Table 3, the content of compiled problems and suggestions could be categorized into 15 aspects covering the whole process of implementing NCDP in medical institutions, which revealed the fact that Chinese medical institutions are facing serious challenges in the implementation of NCDP. Statistical analysis revealed significant differences in the number of relevant problems and suggestions between the 15 categories. The gathered opinions and suggestions were relatively focused on the procurement and supply, reporting of procurement volume, and clinical priority use of security measures of drugs with national centralized procurement in Chinese medical institutions, as evidenced by the number of relevant opinions and suggestions accounted for 15%, 11.40%, and 9.48%, respectively. To illustrate the problems and suggestions under the various themes, the detailed enumeration of collected problems and suggestions is displayed in Table 4; Supplementary Table S2.

**TABLE 3:** Thematic content distribution of problems and suggestions.

Theme	Number (%)
1. The procurement and supply of DNCP in medical institutions	204(15.00)
2. Reasonable measurement and reporting of data related to the procurement volume of DNCP in medical institutions	155(11.40)
3. Safeguards for priority clinical use of DNCP in medical institutions	129(9.48)
4. Catalog construction and management of DNCP in medical institutes	125(9.19)
5. Policy advocacy guidance and risk prevention and control of NCDP	95(6.98)
6. Rational allocation of contract usage of DNCP in medical institutes	92(6.76)
7. Progress monitoring and analysis of contract usage completion of DNCP in medical institutes	87(6.40)
8. Management of clinical rational use of alternative DNCP	78(5.74)
9. Health insurance fund balance retention incentive system of NCDP	76(5.58)
10. Clinical reasonable use assessment system of DNCP in medical institutes	74(5.43)
11. Implementation of DNCP-related system construction in medical institutes	65(4.79)
12. Information technology support system of management of DNCP in medical institutes	53(3.91)
13. The organization system and responsibilities of working group on NCDP in medical institutions	46(3.35)
14. Monitoring and management of quality and adverse event of DNCP	41(3.03)
15. Comprehensive clinical evaluation work of DNCP	40(2.95)
*p* ***-value***	<0.01

χ2 test.

**TABLE 4:** The model sample of collected problems and suggestions in major themes.

**1. Purchasing supply of DNCP**
***P:*** The shortage of supply for winning drugs.
***S:*** The higher authorities, distribution companies and medical institutions are optimally managed in three stages: before, during and after the event.
**2. Rational measurement and reporting of data related to procurement volume**
***P:*** (1) The long procurement cycle and time gap between the reporting of purchase volume and execution resulting in contract usage not being completed as expected.
(2) Clinical use of antimicrobial drugs is affected by several factors, making it more difficult to complete the agreed procurement volume. (3) Specialized hospitals hard to complete the agreed procurement volume of non-specialized drugs.
***S:*** (1) Timely sorting the clinically relevant guidelines, establishing a channel for reporting complaints in the implementation process. (2) Special management drugs allowed to report volume according to actual situation, rather than enforcing the contracted volume. (3) Fully consideration of the special demands for specialized hospitals.
**3. Safeguard measures of clinical priority use for DNCP**
***P:*** DNCP, national essential drugs (NED) and provincial centralized procurement drugs have the control requirements of indicators, which will affect each other during clinical use.
***S:*** (1) Centralized procurement of drugs only carried out by the state. (2) The varieties of drugs centralized procurement at the national and provincial levels should be separated. (3) More NED should be implemented for national centralized procurement, while more DNCP should be included in the catalog of NED. (4) Medical insurance, healthcare and other related departments to strengthen coordination and develop a unified assessment index.
**4. Construction and management of catalog of DNCP in medical institutions**
***P:*** The increase in the number of batches and the different starting times of each batch increase the difficulty of managing the catalog of DNCP in medical institutions.
***S:*** Extend the renewal cycle appropriately and try to align the timing of renewal batches.
**5. Policy advocacy guidance and risk control of DNCP in medical institutions**
***P:*** (1) Some patients and medical professionals refuse to drug replacement for lack confidence in the efficacy and quality of the winning drugs, resulting from the insufficiency of the publicity and guidance on DCNP. (2) The inability of continuously supply for winning drugs has seriously affected the publicity effect.
***S:*** (1) Experts from authoritative departments were invited to popularize and promote science for the public and medical personnel through mainstream newspapers, magazines and new media. (2) National drug supervision and management departments strictly control the quality of DNCP, increase the frequency of DNCP quality sampling and real-time publication of the results. (3) The provincial health insurance bureaus and other relevant departments establish emergency reserves of manufacturers, inventory and discontinuation of production reporting system, for the inability to protect the behavior of drugs to take measures such as compensation, discipline, withdrawal, alternative and emergency security.

Note. ***P***, problems. ***S***, suggestions.

### Correlation between the region of the medical institution and collected problems

In this study, questionnaires were distributed to 329 Chinese medical institutions and collected 1360 problems and suggestions. As displayed in Table 5, there was statistically significant difference in the number of problems and suggestions between regions (*p* < 0.001), indicating that the implementation of NCDP by medical institutions is closely related to the region of the medical institution. Besides, the gathered feedback regarding the procurement and supply of drugs with national centralized procurement displayed significant difference (*p* = 0.0096) between regions (Table 6). Therefore, subsequent policy development and optimization should take regional differences into account.

**TABLE 5:** Regional distribution of collected problems and suggestions.

Regions		Medical institutions	Problems and suggestions	
		Number	Number	Mean
		*n* = 329	*n* = 1360	—
Southwest China		49	416	8.490
East China		110	346	3.145
Northwest China		23	191	8.304
Central China		33	175	5.303
South China		32	103	3.219
North China		63	96	1.524
Northeast China		19	33	1.737
*p* ***-value***		—	—	<0.001

One-way ANOVA, analysis.

**TABLE 6:** Regional distribution of problems in the supply of DNCP.

Regions	Problems and suggestions
—	*n* = 204
Southwest China	66
East China	49
Northwest China	23
Central China	41
South China	15
North China	7
Northeast China	3
*p*-value	0.0096

One-way ANOVA analysis.

## Discussion

NCDP is a unique form of drug procurement in China, which playing a positive role in Chinese healthcare reform through reducing the prices of medicines and improving the rationality of clinical use of medicines. However, NCDP has gradually highlighted a number of typical problems and contradictions over the years, which have directly led to a reduction in the efficiency of the policy (Gong et al., 2021; Zou et al., 2023). In this study, we aimed to reveal the situation of NCDP in Chinese medical institutions. The questionnaires were distributed to 329 Chinese medical institutions from North, Northeast, East, Central, South, Southwest, and Northwest China, which contains provincial, municipal, and community medical institutions. Meanwhile, this survey focused on multidisciplinary medical staff centered on pharmacy staff, which play a key role in the implementation of NCDP in medical institutions. Therefore, this study provides a more comprehensive and in-depth understanding of the current situation of NCDP in Chinese medical institutions. Furthermore, 329 valid questionnaires and 1360 problems and suggestions were collected. These problems and suggestions cover the whole process of implementation of NCDP, indicating the Chinese medical institutions is facing great challenges in the execution of NCDP.

There was statistically significant difference in the number of problems and suggestions between regions. Of these, the medical institutions coming from Southwest and Northwest regions have the highest average number of opinions and suggestions, probably due to the fact that these regions contain more remote areas, making it more difficult to implement the policy. The most suggested topics involve the clinical rational use of DNCP in medical institutions, including six aspects such as reasonable allocation of contract dosage, monitoring and analysis of completion progress, guarantee measures for clinical priority use, totaling 536 articles (39.41%). In addition, a total of 484 recommendations (35.59%) related to the procurement and supply guarantee of DNCP in medical institutions, including the reasonable measurement and reporting of data related to procurement volume, the construction and management of DNCP catalogs, and procurement and supply, which are basically consistent with those reported in the literature (Chinese Pharmacists Association.2023; Zou et al., 2023). The results demonstrated that the rational clinical use and security of supply are still the most important concerns of medical institutions in the process of implementing the NCDP policy. Among the collected opinions and suggestions, the largest number of problems and suggestions are related to DNCP procurement supply, a total of 204, accounting for 15%, focusing on the shortage of supply of the winning drugs. Some medical institutions reflected that most varieties of the winning drugs were in short supply at the beginning of national centralized drug procurement and 1 to 2 months before the end of the procurement cycle, resulting in discontinuity of clinical treatment, and even some winning drugs were in short supply for a long time, such as acarbose tablets, celecoxib capsules and tenofovir disoproxil fumarate tablets (Xu.2022). Remote areas such as Tibet and Xinjiang lack drug manufacturers, and the vast majority of drugs are transported from the mainland (Li, Yan, Bai, Li and Shao.2022). In addition, icy roads in winter make drugs not available in time, which can further cause local drug shortages and stock-outs. At the same time, some drugs are used in remote areas in small quantities and cannot reach the minimum delivery quantity of the manufacturer, which can also lead to abnormal drug supply. These results are mainly the result of three reasons. First of all, there are problems with the production capacity of manufacturers for winning drugs. Some of winning drug manufacturers failed to fully anticipate the inaccuracy of the medical institutions to report the volume or exceed the agreed procurement volume, resulting in an oversupply (Liu.2023; Sun et al., 2023). Secondly, there are problems with distribution enterprises in the transportation of winning drugs (Yang et al., 2023). According to the relevant documents, only one distribution enterprise is allowed for the winning drugs, but some of the distribution enterprises are small in scale with narrow distribution network, which affects the stability of the drug supply. Third, drug manufacturers control the sale of drugs in quantity and distribution. Some of the selected drug manufacturers who have completed the quantitative procurement of the winning drugs have controlled the distribution of drugs, resulting in tight supply or even shortage of drugs.

In order to avoid the above problems, experts from the participating medical institutions suggest optimizing the management involved in the pre, in and post process. For the pre-event phase, the competent authorities in the tender to fully assess the supply capacity of drug manufacturers. The winning drugs have at least two distribution enterprises for medical institutions to choose. By increasing the supervision of drug distribution enterprises to achieve a smooth interface between the drug manufacturer and the distributor (Tan et al., 2020). Because primary medical institutions in remote areas have less demand for drug use, which inevitably reduces the motivation of enterprises to distribute, the distribution rate of drugs in remote areas can be included as part of the qualification audit of distribution enterprises. In addition, medical institutions can make appropriate reserves of non-winning drugs before the start of a new batch of centralized procurement, leaving a buffer period of 1–2 months between procurement cycles. For the in-event phase, the competent departments regularly supervise drug manufacturers and distribution enterprises. Medical institutions establish contingency plans for shortages of DNCP, and report the production and distribution of the selected enterprises and distribution enterprises to the management. Collective drug procurement platform records the selected drug shortage information and vouchers, while the temporary procurement quantity is combined into the agreed procurement volume. For the post-event phase, the regulatory authorities can strengthen the monitoring and evaluation of the selected production enterprises, and include their production capacity, ability to guarantee supply and integrity into the assessment system. At the same time, it is recommended that specific penalty regulations be formulated at the national level to punish the consequences of default such as malicious bidding or inability to complete the procurement schedule after winning a bid, and failure to guarantee product quality after winning a bid. Increasing penalties at the national level can guide enterprises to bid wisely and strengthen the contractual spirit.

## Conclusion

The problems collected in this research cover the whole process of implementing NCDP by medical institutions, and some relatively concentrated problems cannot be solved by medical institutions on their own, which suggests that only continuous summary experience, comparative research and based on national conditions can consolidate and expand the results of NCDP. The deep-seated conflicts in the procurement process should be directly confronted and corresponding solutions proposed in order to achieve meeting the clinical demand for drugs, promoting rational drug management, optimizing hospital management, and forming a market-based price formation mechanism. Only by formulating scientific and reasonable policies, combining and forming synergy from medical insurance, medical treatment, medicine, and patients, can we break through the existing blind spots and blockages, ensure that drug procurement policies are gradually moving on a good and correct track, and realize the reform of the medical and health system to benefit the general public.

## Data Availability

The original contributions presented in the study are included in the article/Supplementary Material, further inquiries can be directed to the corresponding authors.
