# Supplementary material for: Problems and challenges encountered by Chinese medical institutions in implementing the national centralized drug procurement
Source: Front Pharmacol. 2023 Aug 30;14:1233491. doi: 10.3389/fphar.2023.1233491 (PMC10513935; doi:10.3389/fphar.2023.1233491)
Supplement: Supplementary file 1 [file Table1.docx]

Supplementary Material

Problems and challenges encountered by Chinese medical institutions in implementing the national centralized drug procurement

Wen Zhang^1†^, Qingwen Xu^1†^, Jing Peng^1^, Xiaotong Zhang^1^, Lu Chen^1^, Yilai Wu^1^, Kui Yang^1^, Jiajie Luan^1*^, Xiaoyun Liu^1*^

^1^ The First Affiliated Hospital of Wannan Medical College (Yijishan Hospital of Wannan Medical College), Wuhu, Anhui, China

*** Correspondence:** Xiaoyun Liu (20111223@wnmc.edu.cn) and Jiajie Luan (luanjiajie757@163.com)

**†** These authors contributed equally to this work and share first authorship.

# Supplementary Tables

**Supplementary Table 1. Catalogue of Questionnaire**

| **1. Construction of system and organizational system of NCDP in medical institutions**  1.1 The implement of system construction related to NCDP in medical institutions  1.2 The organization system and responsibilities of working group on NCDP in medical institutions  **2. Procurement and supply security of drugs with national centralized procurement (DCNP) in medical institutions**  2.1 Reasonable measurement and reporting of data related to the procurement volume of DCNP by medical institutions  2.2 Construction and management of catalog of DCNP in medical institutions  2.3 Purchasing supply of DCNP in medical institutions  **3. Rational clinical use of national DCNP in medical institutions**  3.1 Reasonable allocation of task volume of DCNP for medical institutions  3.2 Monitoring and analysis of the progress of the completion of the task of DCNP  3.3 Safeguards for priority clinical use of DCNP  3.4 Clinical rational use management of similar alternative drugs for DCNP  3.5 Clinical rational use assessment system for DCNP in medical institutions  3.6 Health insurance fund balance retention incentive system  **4. Comprehensive clinical evaluation of DCNP for medical institutions**  4.1 Monitoring and management of quality and adverse events of DCNP  4.2 Carrying out comprehensive clinical evaluation of DCNP  **5. Information technology support system of DCNP for medical institutions**  **6. Policy advocacy guidance and risk prevention and control of DCNP for medical institutions** |
| --- |

**Supplementary Table 2. The model sample of collected problems and suggestions**

| **1. The allocation of contracted usage of DNCP in medical institutions**  ***P:*** Clinical departments are required to complete the double the contract dosage in the same time period due to the overlap of the time periods of the agreed procurement volume cycle for DNCP with different sizes in same batch and DNCP with different dosage forms in different batches.  ***S:*** The higher authority coordinates the promotion and implementation of the work of each batch of DNCP, clarify the accountability of contract usage, and improve the relevant measures.  **2. Monitoring and analysis of the completion of the contracted usage of DNCP**  ***P:*** It is unable to real-time monitor the completion of the contracted usage of DNCP.  ***S:*** Establish a perfect information technology support system to achieve real-time supervision of the contract dosage of DNCP.  **3. Clinical rational use management of alternative drugs for winning drugs**  ***P:*** Inability to monitor the rational clinical use of alternative drugs in real time.  ***S:*** Establish a perfect information support system for alternative drugs.  **4. Medical insurance fund balance retention incentive system**  ***P:*** (1) Late disbursement of medical insurance balance retention funds (MIBRF). (2) The allocated MIBRF are not sufficient to cover the expenditures of the management of the DNCP in medical institutions. (3) Local management of MIBRF in some area make hard to cash in on balance retention for off-site medical patients.  (4) The local health insurance policy of some DRG payment pilot medical institutions stipulates that no health insurance fund balance retention rebate payment will be given.  ***S:*** (1) Medical institutions related to medical insurance funds and balance retention money should be allocated in a timely manner, and a scientific and reasonable way of calculating the balance retention funds should be determined after taking into full consideration factors such as off-site medical insurance. (2) The higher authorities should impose penalties on the relevant departments and individuals who default on the retention of medical institution balances without justifiable reasons.  **5. Assessment system of clinical rational use for DNCP in medical institutions**  ***P:*** A single assessment index cannot assess the rational use of DNCP.  ***S:*** Establish a scientific assessment system for clinical rational use of DNCP.  **6. Related system construction**  ***P*:** The system or program is still not comprehensive and complete.  ***S*:** Medical institutions should establish the corresponding management system.  **7. Information technology support system for DNCP in medical institutions**  ***P:*** (1) Information construction level of medical institutions in each region varies greatly. (2) There is a big gap in the application level of information technology in each medical institution. (3) Most medical institutions information systems are still stuck in His system identification and summary of purchase volume, and most of the statistical work is done manually.  ***S:*** (1) Special funds reserved for medical insurance fund balance could be used to build the technical support system of management of DNCP in medical institutions. (2) The higher authorities should do homogeneous management and guidance for the information management of DNCP.  **8. Organizational system and job responsibilities for working group**  ***P****:* The pharmacy department, undertaking core work of NCDP, lacks the ability to mobilize and coordinate with other departments.  ***S*:** A working group led by departments with administrative intervention powers to coordinate the planning and supervision of the management of DNCP.  **9. Monitoring and management of quality and adverse events of DNCP**  ***P:*** There is no separate channel for reporting adverse reactions of DNCP, and the Chinese pharmacovigilance system is temporarily unable to achieve variety monitoring, special monitoring and self-monitoring, and there is no real-time update function.  ***S:*** Monitoring of adverse events of DNCP should be carried out.  **10. Comprehensive clinical evaluation of DNCP**  ***P:*** (1) The lack of data on clinical efficacy and adverse reactions caused the poor clinical acceptance of some winning drugs, which passed consistent evaluation. (2) The low acceptance of DNCP for special populations including patients with mental illnesses, cancer patients (complimentary drug model), and patients with acute illnesses has led to more related complaints and disputes.  ***S:*** (1) The higher authorities conduct research on the quality of DNCP and increase publicity. (2) Carry out comprehensive clinical evaluation of DNCP according to the principles of standardization, scientific and homogenization. |
| --- |

Note. ***P***, Problems. ***S***, Suggestions.
